# Replacing carbohydrate with protein and fat in prediabetes or type-2 diabetes: greater effect on metabolites in PBMC than plasma

**DOI:** 10.1186/s12986-016-0063-4

**Published:** 2016-01-19

**Authors:** Minjoo Kim, Gayoung Song, Miso Kang, Hye Jin Yoo, Tae-Sook Jeong, Sang-Hyun Lee, Jong Ho Lee

**Affiliations:** Research Center for Silver Science, Institute of Symbiotic Life-TECH, Yonsei University, Seoul, 03722 Republic of Korea; National Leading Research Laboratory of Clinical Nutrigenetics/Nutrigenomics, Department of Food and Nutrition, College of Human Ecology, Yonsei University, Seoul, 03722 Republic of Korea; Department of Food and Nutrition, Brain Korea 21 PLUS Project, College of Human Ecology, Yonsei University, 50 Yonsei-ro, Seodaemun-gu, Seoul, 03722 Republic of Korea; National Research Laboratory of Lipid Metabolism and Atherosclerosis, Korea Research Institute of Bioscience and Biotechnology, Daejeon, 34141 Republic of Korea; Department of Family Practice, National Health Insurance Corporation Ilsan Hospital, Goyang, 10444 Republic of Korea

**Keywords:** Metabolites, Peripheral blood mononuclear cells, Prediabetes, Whole-grains and legumes

## Abstract

**Background:**

Active metabolism of peripheral blood mononuclear cells (PBMC) could suggest their suitability for metabolomics studies. This study examined whether reductions in PBMCs and plasma lipoprotein-associated phospholipase A_2_ (Lp-PLA_2_) activities induced by dietary intervention affected the overall metabolic profiles of PBMC and plasma.

**Methods:**

Eighty nonobese subjects aged 40–70 years (18.5 ≤ BMI < 30 kg/m^2^) with prediabetes or newly-diagnosed type-2 diabetes were assigned to consume either the usual refined-rice diet (control group, n = 40) or to replace refined rice with whole grains and legumes as carbohydrates (whole-grain group, n = 40) for three meals per day during the 12-week intervention. Fasting PBMC and plasma metabolomes were profiled using UPLC-LTQ-Orbitrap mass spectrometry.

**Results:**

After 12 weeks, changes in fasting glucose, HbA_1c_, HOMA-IR, MDA, ox-LDL, LDL particle size, plasma Lp-PLA_2_ activity, and PBMC enzyme activity in the whole-grain group were significantly different from those in the control group before and after adjusting for baseline levels. The PBMC levels of L-leucine, oleamide, lysoPC (16:0), and lysoPC (18:0) in the whole-grain group showed greater reductions compared with those of the control group. Changes in plasma metabolites were not significantly different between the two groups. Changes in PBMC Lp-PLA_2_ activity positively correlated with changes in L-leucine, oleamide, lysoPC (16:0), lysoPC (18:0), glucose, and ox-LDL, and negatively correlated with changes in LDL particle size.

**Conclusions:**

This study showed that dietary intervention in prediabetic or type-2 diabetic patients had a greater effect on PBMC Lp-PLA_2_ activity and metabolites compared with those of plasma metabolites.

**Trial registration:**

NCT02191644

## Background

Diabetes is an epidemic metabolic disorder; about 2.7 million Korean people (8.03 %) aged 30 years or older had type-2 diabetes (T2D) and 25.0 % of adults had prediabetes in 2013 according to Korean Diabetes Fact Sheet 2015. Diabetes-related mortality was steadily decreased since 2003 and ranked as the fifth leading cause of natural death [1]. Prediabetes can be indicated by either impaired fasting glucose (IFG) by the American Diabetes Association criteria [2] or impaired glucose tolerance (IGT) by World Health Organization criteria [3].

Lipoprotein-associated phospholipase A_2_ (Lp-PLA_2_) independently predicts T2D incidence and may be involved in its etiology [4]. In recent study, an inverse association was observed between protein intake and circulating Lp-PLA_2_ activity, suggesting that nutritional factors may influence Lp-PLA_2_ activity [5]. An intervention study that replaced refined rice with whole grains and legumes reduced blood glucose, insulin, Lp-PLA_2_ activity, and cardiovascular risk factors in patients with prediabetes or T2D [6]. The effects of this intervention diet on plasma and peripheral blood mononuclear cell (PBMC) metabolites have not been determined.

PBMCs include monocytes and lymphocytes which are blood cells having a round nucleus. These blood cells are a critical component in the immune system to fight infection and adapt to intruders. Monocytes have a key role in onset and development of inflammatory reactions by generating bioactive molecules such as Lp-PLA_2_ in response to inflammatory stimuli [7]. Lymphocytes are consist of three major types; T cells, B cells, and natural killer cells. T cells and B cells are the major cellular components of the adaptive immune response, whereas natural killer cells are a part of the innate immune system. The production and release of Lp-PLA_2_ by lymphocytes may become increased under inflammatory conditions [8]. Dietary intervention induces PBMC gene expression changes, including downregulating genes involved in inflammatory processes [9]. Therefore, changes in PBMC metabolites and Lp-PLA_2_ activity after dietary intervention could reflect dynamic responses, which are not detectable in plasma metabolomics analyses. The aim of this 12-week intervention study was to examine whether reductions in PBMC and plasma Lp-PLA_2_ activities induced by dietary intervention (replacement of refined rice with whole grains and legumes, and higher intake of vegetables) affected the overall metabolic profiles of PBMC and plasma in nonobese patients that exhibited IFG, IGT, or newly-diagnosed T2D.

## Methods

### Subjects and study design

Nonobese subjects aged 40–70 years (18.5 ≤ BMI < 30 kg/m^2^) were recruited from the Health Service Center (HSC) at the Ilsan Hospital, Goyang, Korea, during January–June 2013. Based on the HSC data, subjects who had IFG (100 ≤ fasting glucose <126 mg/dL) or newly-diagnosed T2D (fasting glucose ≥126 mg/dL) were referred to the Department of Family Medicine or Internal Medicine. Exclusion criteria included: current and/or past history of cardiovascular disease; liver or kidney dysfunction; thyroid or pituitary disease. Subjects who were taking medications or supplements also were excluded. A total of 82 subjects were enrolled. The macronutrient composition of each subject’s usual diet corresponded to a typical diet with cooked refined rice. The purpose of the study was carefully explained to all participants, and written consent was obtained prior to their participation. The Institutional Review Board of the NHIC-sponsored Ilsan Hospital and Yonsei University provided ethical approval of the study protocol, which was performed according to the Helsinki Declaration.

The present study was performed in two phases, including a 2-week run-in phase consisting of the usual diet with refined rice, and a 12-week intervention phase. During the run-in period, two subjects who did not maintain their energy intake dropped out. The remaining 80 subjects were randomly subdivided into the two study groups, and were assigned to consume either the usual refined-rice diet (control group, n = 40) or to replace refined rice with whole grains and legumes as carbohydrates (whole-grain group, n = 40) for three meals per day during the 12-week intervention.

### Assessment of dietary intake and physical activity level

All subjects were given written and verbal instructions by a registered dietitian on completion of a 3-day (2 week days and 1 weekend day) dietary record every 2 weeks throughout the study. On the dietary record sheet, subjects were instructed to weigh and record the food amount before and after ingestion. All participants were advised to continue their usual refined-rice diet during a 2-week run-in period. Baseline measurements were performed at the start of the run-in phase. After a run-in period, subjects in the control group maintained the usual refined-rice diet, whereas subjects in the whole-grain group replaced refined rice with a mix of 1/3 legumes, 1/3 barley, and 1/3 wild rice three times per day, and increased vegetable intake to at least 6 units (30–70 g/unit) per day for sufficient dietary fiber intake. The dietitian monitored subject compliance and body-weight changes during the whole study by performing biweekly visits or telephone interviews and all participants were encouraged to maintain their usual lifestyles. Dietary energy values and nutrient contents from 3-day food records were calculated using the CAN-pro 3.0 (Korean Nutrition Society, Seoul, Korea). Total energy expenditures (kcal/day) were calculated from activity patterns including basal metabolic rate, physical activity for 24 h [10], and specific food dynamic action. Basal metabolic rate for each subject was calculated with the Harris–Benedict equation [11].

### Anthropometry and blood pressure analysis

Body weight and height of unclothed subjects without shoes were measured in the morning for calculating body mass index (BMI, kg/m^2^). Waist circumference was measured on standing subjects at the umbilical level after normal expiration. Blood pressure (BP) of seated subjects after a 20-min rest was measured in the left arm with an automatic BP monitor (FT-200S, Jawon Medical, Gyeongsan, Korea). After a 12-h fasting period, venous blood specimens were collected in EDTA-treated and plain tubes and centrifuged to yield plasma or serum, respectively, which were stored at −70 °C until analysis.

### Clinical measurements

Fasting total cholesterol and triglyceride levels were analyzed using a Hitachi 7600 Autoanalyzer (Hitachi Ltd., Tokyo, Japan). ApoB-containing lipoproteins were precipitated with dextran-magnesium sulfate, and high density lipoprotein (HDL)-cholesterol concentrations in patient serum samples were measured enzymatically. For subjects with serum triglyceride levels <400 mg/dL, low density lipoprotein (LDL)-cholesterol concentrations were estimated indirectly using the Friedewald formula. For subjects with serum triglyceride levels ≥400 mg/dL, LDL-cholesterol concentrations were measured directly. Free fatty acids (FFA) were analyzed using the acyl-CoA synthetase − acyl-CoA oxidase enzymatic assay method and a Hitachi 7600 Autoanalyzer.

All subjects underwent an oral glucose-tolerance test at 0 and 12 weeks by ingesting a 75 g glucose solution after a 12-h overnight fast. Venous specimens were collected before glucose loading, at loading, and 30, 60, and 120-min after loading to determine serum glucose levels and responses. Fasting glucose levels were analyzed by the hexokinase method using a Hitachi 7600 Autoanalyzer. Insulin levels were measured using an immunoradiometric assay kit from DIAsource ImmunoAssays S.A. (Louvain, Belgium). Hemoglobin A_1c_ (HbA_1c_) was measured by immonoturbidimetric analysis. Insulin resistance (IR) was calculated by the homeostasis-model assessment (HOMA) [12].

Whole blood was mixed with the same volume of RPMI 1640 (Gibco, Life Technologies, Gland Island, NY) and gently laid on a histopaque-1077 (Sigma-Aldrich, St. Louis, MO). The sample was then centrifuged at 1800 rpm for 20-min at 10 °C. After the separation, a thin layer of PBMCs was isolated and washed twice with RPMI 1640. The pellet was resuspended in RPMI 1640 with streptomycin. Isolated PBMCs were cultured in RPMI 1640 supplemented with 10 % fetal bovine serum (FBS), seeded in 12-well plates (1x10^6^ cells/mL; SPL, Gyeonggi-do, Korea), and incubated at 37 °C with 5 % CO_2_ for 22.5 h. After a 22.5 h incubation, 10 % FBS was added and incubated for 24.5 h. At third day, PBMC supernatants were collected and stored at −80 °C until Lp-PLA_2_ activity levels were assayed. Lp-PLA_2_ activity in plasma and PBMC supernatants was measured by using a modification of a previously described high-throughput radiometric activity assay [13].

Serum high-sensitivity C-reactive protein (hs-CRP) was measured with an ADVIA 2400 Clinical Chemistry System (Siemens Ltd., Tarrytown, NY) using a commercially available, hs-CRP-Latex(II) *X*2 kit (Denka-Seiken Co., Ltd., Tokyo, Japan). Plasma malondialdehyde (MDA) was measured from thiobarbituric acid − reactive substances (TBARS) using the TBARS Assay Kit (ZeptoMetrix Co., Buffalo, NY). LDL particles were isolated by sequential flotation ultracentrifugation, and particle size distribution (1.019–1.063 g/mL) was examined using a pore-gradient lipoprotein system (CBS Scientific Company, San Diego, CA) on commercially available, non-denaturing gels containing a linear 2–16 % acrylamide gradient (CBS Scientific Company). Latex-bead (30 nm) conjugated thyroglobulin (17 nm), ferritin (12.2 nm), and catalase (10.4 nm) standards were used to estimate the relative band migration rates. Gels were scanned using a GS-800 Calibrated Imaging Densitometer (Bio-Rad Laboratories, Hercules, CA). Plasma oxidized (ox)-LDL was measured using an enzyme immunoassay (Mercodia AB, Uppsala, Sweden), and the resulting color reaction was determined at 450 nm on a Wallac Victor^2^ multilabel counter (Perkin-Elmer Life Sciences, Boston, MA).

### Global (nontargeted) metabolic profiling of PBMC and plasma

#### PBMC and plasma extract sample preparation

Before analysis, 800 μL of 80 % acetonitrile was added to 100 μL of PBMC and plasma, mixed by vortexing, and centrifuged at 10,000 rpm for 5-min at 4 °C. The supernatant was dried with N_2_ (l), dissolved in 10 % methanol, mixed by vortexing, and centrifuged at 10,000 rpm for 5-min at 4 °C. The supernatant was transferred into a vial.

#### Ultra performance liquid chromatography

PBMC and Plasma extract samples (4 μL) were injected into an Acquity UPLC-BEH-C18 column (2.1 × 50 mm, 1.7 μm; Waters, Milford, MA) that was coupled in-line with a UPLC-LTQ-Orbitrap XL (Thermo Fisher Scientific, Waltham, MA). The injected samples were equilibrated with water containing 0.1 % formic acid. Samples were eluted with an acetonitrile gradient containing 0.1 % formic acid at a flow rate of 0.35 mL/min for 20-min. Metabolites were separated by UPLC, analyzed, and assigned by LTQ-Orbitrap-XL. The mass spectrometer (MS) was operated in ESI-positive mode. The spray voltage was 5 kV. The flow-rate nitrogen sheath gas and the auxiliary gas were 50 and 5 (arbitrary units). The capillary voltage (V), tube-lens voltage (V), and capillary temperature (°C) were kept constant at 35 V, 80 V, and 370 °C. Orbitrap data were collected in the range of *m*/*z* 50–1,000. MS/MS spectra of metabolites were obtained by a collision-energy ramp from 55–65 eV, and conducted with Xcalibur 2.1 and MS Frontier software (Thermo Fisher Scientific).

#### Data processing and identification of metabolites

All MS data including retention times, *m*/*z*, and ion intensities were extracted by SIEVE software (Thermo Fisher Scientific) incorporated into the instrument, and the resulting MS data were assembled into a matrix. SIEVE parameters were set as follows: *m*/*z* range 50–1,000; *m*/*z* width 0.02; retention time width 2.5; and *m*/*z* tolerance 0.005. Metabolites were searched using the following databases: ChemSpider (www.chemspider.com), Human Metabolome (www.hmdb.ca), Lipid MAPS (www.lipidmaps.org), KEGG (www.genome.jp/kegg), and MassBank (www.massbank.jp). Selected metabolites were confirmed by retention times and mass spectra of standard samples.

### Statistical analyses

Statistical analyses were performed using SPSS v. 21.0 (IBM SPSS Statistics 21, Chicago, IL). Skewed variables were logarithmically transformed for statistical analyses. A two-tailed *P*-value of <0.05 was considered statistically significant. Differences in biochemical variables between two groups at baseline and follow-up were tested using Student’s independent *t*-test. General linear model tests were applied to compare parameter changes between the two groups by adjusting for baseline values. Paired *t*-tests were used to evaluate differences between baseline and follow-up levels in each group. Pearson’s and partial correlation coefficients were used to examine the relationships between variables over time. False discovery rate − corrected *q*-values were computed using the R package ‘fdrtool’. Heat map was created to visualize and evaluate correlations among metabolites and conventional risk factors in study populations.

Multivariate statistical analysis was performed using SIMCA-P+ software version 12.0 (Umetrics, Umeå, Sweden). Partial least-squares discriminant analysis (PLS-DA) was used as the classification method for modeling the discrimination between groups by visualizing the score scatter plot or *S*-plot using the first and second PLS components. The goodness-of-fit was quantified by *R*^2^*Y*, whereas the predictive ability was quantified by *Q*^2^*Y*. Generally, *R*^2^*Y* describes how well the data in the training set were mathematically reproduced and varied between 0 and 1 (a value of 1 indicated a model with a perfect fit). Models with *Q*^2^*Y* ≥0.5 were considered to have good predictive capabilities.

## Results

### Clinical characteristics, lipid profiles, and nutrient intake

There were no significant differences between two groups in baseline characteristics including age, gender, smoking, and drinking (data not shown). At baseline, there were no significant differences between two groups in BMI, waist:hip ratio (WHR), systolic BP, diastolic BP, serum triglyceride, total cholesterol, LDL-cholesterol, HDL-cholesterol, FFA, and hs-CRP. BMI, WHR, BP, serum lipid profiles, hs-CRP, total energy expenditure, and total energy intake were similar before and after the study in both groups (data not shown).

Replacement with whole grains and legumes caused significant increase in percent energy intake of protein and fat, and significant decrease in percent energy intake of carbohydrate. The percent energy intake of protein, fat, and carbohydrate significantly differed between the two groups before adjusting for baseline values. The whole-grain group had significant increases in fiber intake and polyunsaturated-to-saturated fatty acids ratio compared with baseline values. After 12-week, the whole-grain group had lower percent energy of carbohydrate, higher percent calorie of protein and fat, and fiber intake than control group (Table 1).

**Table 1:** Biochemical characteristics and estimates of daily nutrient intake before and after 12-week dietary intervention

	Control group (*n* = 40)		Whole-grain group (*n* = 40)		*P* ^a^	*P* ^b^	*P* ^c^	*P* ^d^
	Baseline	Follow-up	Baseline	Follow-up				
Glucose (mg/dL)^*∮*^	119.8 ± 5.40	126.6 ± 6.02^***^	119.4 ± 5.31	110.0 ± 4.58^***^	0.958	0.005		
Change	6.78 ± 1.51		−9.45 ± 1.43				<0.001	<0.001
Glucose AUC (mg/dL × h)^*∮*^	376.8 ± 17.7	390.4 ± 22.9	376.6 ± 18.9	352.0 ± 17.5^*^	0.923	0.225		
Change	13.6 ± 9.52		−24.7 ± 8.75				0.004	0.004
HbA_1c_ (%)^*∮*^	6.46 ± 0.19	6.60 ± 0.19^*^	6.41 ± 0.18	6.18 ± 0.15^*^	0.821	0.061		
Change	0.14 ± 0.06		−0.23 ± 0.12				0.007	0.003
HOMA-IR^*∮*^	2.24 ± 0.15	2.34 ± 0.17	2.18 ± 0.12	1.79 ± 0.12^***^	0.791	0.006		
Change	0.10 ± 0.11		−0.39 ± 0.10				0.002	0.001
Insulin (μIU/mL)^*∮*^	7.58 ± 0.35	7.47 ± 0.43	7.58 ± 0.43	6.72 ± 0.46^**^	0.798	0.132		
Malondialdehyde (nmol/mL)^*∮*^	9.24 ± 0.53	10.2 ± 0.61^***^	9.41 ± 0.39	8.70 ± 0.37^**^	0.530	0.041		
Change	0.98 ± 0.24		−0.71 ± 0.24				0.001	<0.001
Oxidized LDL (U/L)^*∮*^	49.1 ± 2.14	50.7 ± 2.26	48.2 ± 2.01	43.8 ± 2.00^***^	0.846	0.036		
Change	1.65 ± 0.90		−4.41 ± 0.82				0.001	<0.001
LDL particle size (nm)^*∮*^	24.1 ± 0.12	24.1 ± 0.13	24.4 ± 0.15	24.6 ± 0.17^***^	0.214	0.015		
Change	−0.05 ± 0.06		0.24 ± 0.05				0.001	0.001
Plasma Lp-PLA_2_ activity (nmol/mL/min)^*∮*^	30.1 ± 1.64	30.3 ± 1.61	28.0 ± 1.20	25.7 ± 1.11	0.874	0.701		
Change	0.24 ± 0.41		−2.38 ± 0.55				0.001	<0.001
Unstimulated PBMC Lp-PLA_2_ activity (nmol/mL/min)^*∮*^	2.00 ± 0.12	2.28 ± 0.13^**^	2.16 ± 0.12	1.90 ± 0.12^**^	0.365	0.046		
Change	0.28 ± 0.08		−0.26 ± 0.10				0.001	<0.001
Estimate of daily nutrient intake								
Energy intake (kcal/d)	2,143 ± 40	2,170 ± 38	2,166 ± 36	2,185 ± 38	0.665	0.786		
Carbohydrate (%)	61.9 ± 0.16	61.9 ± 0.17	61.9 ± 0.17	54.6 ± 0.11	0.833	<0.001		
Protein (%)	16.3 ± 0.10	16.3 ± 0.08	16.3 ± 0.10	20.5 ± 0.13	0.851	<0.001		
Fat (%)	22.1 ± 0.16	22.2 ± 0.15	22.1 ± 0.16	24.9 ± 0.15	0.731	<0.001		
Crude fiber (g)^*∮*^	25.2 ± 1.20	22.8 ± 1.19	24.5 ± 1.21	28.4 ± 1.18	0.713	0.001		
PUFA/SFA	2.10 ± 0.18	2.10 ± 0.16	2.29 ± 0.16	2.98 ± 0.17	0.418	<0.001		

Mean ± SEM.^∮^tested by logarithmic transformation, *P* ^*a*^, values derived from independent *t*-test in baseline. *P* ^*b*^, values derived from independent *t*-test in follow-up. *P* ^*c*^, values derived from independent *t*-test in changed value. *P* ^*d*^, values derived from independent *t*-test in changed value after adjusting for baseline. ^*^ *P* < 0.05, ^**^ *P* < 0.01, ^***^ *P* < 0.001 derived from paired *t*-test. PBMC, peripheral blood mononuclear cell; AUC, area under the curve; PUFA/SFA, polyunsaturated-to-saturated fatty acids ratio

### Fasting glucose, insulin, and malondialdehyde

At the end of the study, glucose, HbA_1c_, and MDA concentrations significantly increased in the control group, whereas glucose, glucose AUC (area under the curve), HbA_1c_, HOMA-IR, insulin, and MDA significantly decreased in the whole-grain group (Table 1). Changes in glucose, glucose AUC, HbA_1c_, HOMA-IR, and MDA in the whole-grain group were significantly different from those in the control group before and after adjusting for baseline levels. Post-treatment glucose, HOMA-IR, and MDA in the whole-grain group were significantly lower than those in the control group (Table 1).

### Plasma ox-LDL, LDL particle size, Lp-PLA_2_ activity in plasma and unstimulated PBMC

At the end of the study, the whole-grain group had lower ox-LDL and Lp-PLA_2_ activity in PBMC and larger LDL particle size, whereas the control group had higher Lp-PLA_2_ activity in PBMC (Table 1). These changes in ox-LDL, LDL particle size, plasma Lp-PLA_2_ activity, and PBMC Lp-PLA_2_ activity in the whole-grain group were significantly different from those in the control group before and after adjusting for baseline levels. The post-treatment whole-grain group had lower ox-LDL and PBMC Lp-PLA_2_ activity, and larger LDL particle size than control group (Table 1).

### Metabolic profiling of PBMC and plasma using UPLC-LTQ-orbitrap MS

#### Nontargeted metabolic pattern analysis

**Fig. 1:**
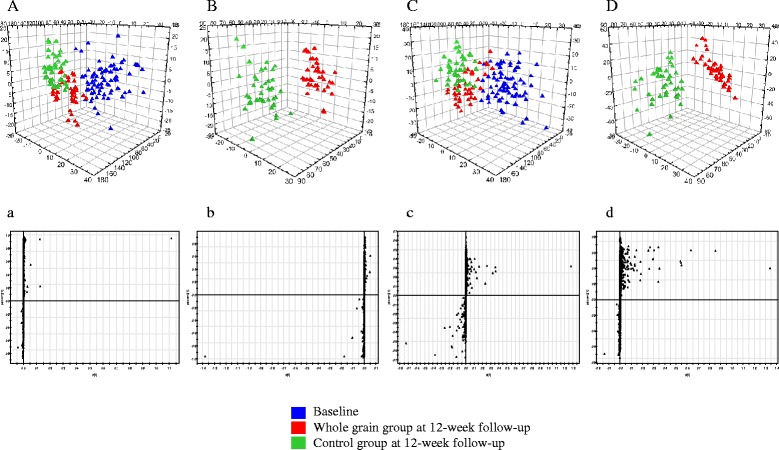
Identification of PBMC and plasma metabolites that were significantly altered at 12-week follow-up. **a** Partial least squares discriminant analysis score scatter plot (PLS-DA score scatter plot) of PBMC metabolites at baseline (*n* = 80), whole-grain group at follow-up (*n* = 40), and control group at follow-up (*n* = 40). **b** PLS-DA score scatter plot of PBMC metabolites for whole-grain group at follow-up (*n* = 40) and control group at follow-up (*n* = 40). **c** PLS-DA PLS-DA score scatter plot of plasma metabolites at baseline (*n* = 80), whole-grain group at follow-up (*n* = 40), and control group at follow-up (*n* = 40). **d** PLS-DA score scatter plot of plasma metabolites for whole-grain group at follow-up (*n* = 40) and control group at follow-up (*n* = 40)

MS data of PBMC and plasma metabolites obtained at baseline and follow-up were analyzed with PLS-DA score scatter plot for the following two combinations: 1) control and whole-grain groups at baseline, control group at follow-up, and whole-grain group at follow-up (Fig. 1a, PBMC; Fig. 1c, plasma); and 2) control and whole-grain groups at follow-up (Fig. 1b, PBMC; Fig. 1d, plasma). The PBMC metabolite PLS-DA score scatter plot showed distinct clustering and clear separation for the following subjects: control and whole-grain groups at baseline, control group at follow-up, and whole-grain group at follow-up [*R*^2^*X*(cum) = 0.124, *R*^2^*Y*(cum) = 0.34, *Q*^2^*Y*(cum) = 0.218] (Fig. 1a). These distinct clusters indicate that PBMC profiling detects metabolic changes induced by dietary intervention. The PBMC metabolite PLS-DA score scatter plot showed distinct clustering for control and whole-grain groups at follow-up [*R*^*2*^*X*(cum) = 0.125, *R*^*2*^*Y*(cum) = 0.809, *Q*^*2*^*Y*(cum) = 0.525] (Fig. 1b).

The plasma metabolite PLS-DA score scatter plot were not as clearly clustered as those for PBMC metabolites [*R*^2^*X*(cum) = 0.124, *R*^2^*Y*(cum) = 0.376, *Q*^2^*Y*(cum) = 0.127] (Fig. 1c). For control and whole-grain groups at follow-up, the plasma metabolite PLS-DA score scatter plot were not as clearly clustered as those for PBMC metabolites [*R*^*2*^*X*(cum) = 0.201, *R*^*2*^*Y*(cum) = 0.704, *Q*^*2*^*Y*(cum) = 0.414] (Fig. 1d). To identify metabolites that differentially determined data at baseline and follow-up, *S*-plots of p(1) and p(corr) (1) were generated using centroid scaling. The *S*-plots revealed that metabolites with higher or lower p(corr) values more clearly discriminated between the two groups.

#### Identification of PBMC metabolites

Of 1,923 PBMC metabolites, those that correlated with separation between the groups were identified by the variable important in the projection (VIP) parameter; VIP values >1.0 were highly relevant for group differences. 51 metabolites had VIP >1.0; 10 of these were previously identified and 41 were unknown. Those 10 PBMC metabolites at baseline and follow-up are shown in Table 2. There were no significant differences in baseline metabolites between two groups. After follow-up, the control group showed significant changes in six PBMC metabolite levels, whereas the whole-grain group showed significant changes in seven PBMC metabolite levels (Table 2).

**Table 2:** Identification of PBMC metabolites at baseline and 12-week follow-up

Metabolite	Formula [M + H]^+^	Exact mass (M + H)	Normalized peak intensity				Variable important in the projection		
			Control group (*n* = 40)		Whole-grain group (*n* = 40)		Baseline vs. follow-up		12-weeks
			Baseline	Follow-up	Baseline	Follow-up	Control	Whole-grain	Control vs. whole-grain
Cyclopentanone dimethylhydrazone	C_7_H_14_N_2_	127.1235	82,551 ± 21,507	101,931 ± 26,693^*^	43,192 ± 11,728	51,504 ± 14,505^*^	0.0722	0.1523	1.0119
L-Pyroglutamic acid	C_5_H_7_NO_3_	130.0504	2,410,045 ± 108,001	2,075,511 ± 48,362^*^	2,455,521 ± 70,667	2,484,381 ± 103,907^*††*^	0.1241	3.9771	2.6550
L-Leucine	C_6_H_13_NO_2_	132.1025	7,884,178 ± 309,297	7,922,462 ± 251,819	8,253,845 ± 170,803	7,531,331 ± 171,144^**^	1.8209	3.1661	5.0363
Change			38,283 ± 198,486		−722,514 ± 145,341^*‡*^				
L-Phenylalanine	C_9_H_11_NO_2_	166.0868	2,880,509 ± 113,266	2,755,872 ± 75,646	2,825,355 ± 101,371	2,761,323 ± 56,360	0.5962	0.6567	1.1474
Dihydrobiopterin	C_9_H_13_N5O_3_	239.1018	432,360 ± 23,845	335,279 ± 14,924^***^	427,487 ± 16,797	383,280 ± 15,727^*^	0.4537	1.2616	0.4680
Palmitic amide	C_16_H_33_NO	256.2640	354,492 ± 50,930	330,909 ± 44,809	490,272 ± 84,177	172,851 ± 26,551^**,†^	2.7281	0.1468	2.0394
Ribothymidine	C_10_H_14_N_2_O_6_	258.0930	302,320 ± 22,108	239,902 ± 12,851^**^	322,971 ± 16,498	311,114 ± 19,898^*†*^	0.0730	0.5966	1.3107
Oleamide	C_18_H_35_NO	282.2797	2,676,408 ± 412,881	3,257,688 ± 511,380	3,697,245 ± 621,193	1,491,246 ± 235,475^*,†^	18.4793	8.3202	22.5845
Change			581,279 ± 634,410		−2,205,998 ± 651,404^*‡*^				
LysoPC(16:0)	C_24_H_50_NO_7_P	496.3403	414,438 ± 44,544	645,199 ± 57,544^*^	549,155 ± 59,016	383,399 ± 35,718^*,††^	2.7864	2.8925	3.5066
Change			230,762 ± 72,860		−165,756 ± 63,955^*‡‡*^				
LysoPC(18:0)	C_26_H_54_NO_7_P	524.3716	285,044 ± 26,648	423,718 ± 34,129^**^	364,186 ± 33,732	262,585 ± 23,409^*,††^	1.5197	1.7796	2.0579
Change			138,674 ± 40,222		−101,601 ± 42,602^*‡‡*^				

Mean ± SEM. ^*^ *q* < 0.05, ^**^ *q* < 0.01, ^***^ *q* < 0.001 derived from paired *t*-test. ^*†*^ *q* < 0.05, ^*††*^ *q* < 0.01, ^*†††*^ *q* < 0.001 derived from independent *t*-test in follow-up. ^*‡*^ *q* < 0.05, ^*‡‡*^ *q* < 0.01, ^*‡‡‡*^ *q* < 0.001 derived from changed values between control and whole-grain groups

We compared PBMC metabolite changes between two groups. The whole-grain group had greater reductions in L-leucine (*q* = 0.031), oleamide (*q =* 0.032), lysoPC (16:0) (*q =* 0.003), and lysoPC (18:0) (*q =* 0.003) (Table 2). At follow-up, the whole-grain group had higher peak intensities of L-pyroglutamic acid and ribothymidine, and lower peak intensities of palmitic amide, oleamide, and lysoPCs, compared with those of the control group (Table 2).

#### Identification of plasma metabolites

Of 4,121 plasma metabolites, those that correlated with separation between the groups were selected by VIP >1.0. 122 plasma metabolites were selected; 20 were previously identified and 102 were unknown (Table 3). There were no significant differences in baseline between two groups. After follow-up, C17 sphinganine significantly increased in the control group, also, there were no significant differences in metabolites between two groups, and no significant differences in metabolite changes with respect to baseline (Table 3).

**Table 3:** Identification of plasma metabolites at baseline and 12-week follow-up

Metabolite	Formula [M + H]^+^	Exact mass (M + H)	Normalized peak intensity				Variable important in the projection		
			Control group (*n* = 40)		Whole-grain group (*n* = 40)		Baseline vs. follow-up		12-weeks
			Baseline	Follow-up	Baseline	Follow-up	Control	Whole-grain	Control vs. whole grain
L-Valine	C_5_H_11_NO_2_	118.0868	1,338,114 ± 39,666	1,323,676 ± 36,213	1,328,470 ± 41,793	1,376,655 ± 34,768	0.7807	0.1901	1.0144
L-Leucine	C_6_H_13_NO_2_	132.1025	3,130,607 ± 99,839	3,098,031 ± 98,593	3,130,777 ± 107,368	3,166,315 ± 85,278	0.4904	0.5781	0.7853
L-Phenylalanine	C_9_H_11_NO_2_	166.0868	1,984,169 ± 49,821	1,914,567 ± 45,393	1,997,467 ± 84,582	1,975,319 ± 41,491	0.6137	1.1072	0.7382
Oleamide	C_18_H_35_NO	282.2797	11,074,949 ± 815,519	11,182,173 ± 1,054,856	11,348,184 ± 805,164	12,102,398 ± 709,965	8.4316	19.2720	6.6270
C17 Sphinganine	C_17_H_37_NO_2_	288.2903	323,013 ± 20,549	441,137 ± 24,748^*^	333,258 ± 29,775	355,950 ± 25,250	0.9135	1.3462	0.1114
(4E,8E,10E-d18:3) Sphingosine	C_18_H_33_NO_2_	296.2590	420,731 ± 59,507	832,979 ± 152,996	608,061 ± 81,037	754,737 ± 66,758	1.5120	8.8050	2.2736
Anandamide (18:4, n-3)	C_20_H_33_NO_2_	320.2590	170,194 ± 28,584	237,343 ± 31,518	209,059 ± 26,262	213,643 ± 16,101	0.1156	1.4954	0.5840
LysoPC (14:0)	C_22_H_46_NO_7_P	468.3090	480,474 ± 33,898	503,684 ± 38,015	625,962 ± 45,904	537,767 ± 45,983	1.5352	0.7142	0.2242
LysoPC (16:1)	C_24_H_48_NO_7_P	494.3247	1,133,250 ± 77,052	1,033,013 ± 69,288	1,261,066 ± 78,064	1,127,811 ± 88,549	2.3243	1.4177	1.0354
LysoPC (16:0)	C_24_H_50_NO_7_P	496.3403	15,953,932 ± 573,566	15,910,457 ± 684,453	16,830,932 ± 686,817	15,967,767 ± 537,944	15.3974	7.7607	4.7470
LysoPC (17:0)	C_25_H_52_NO_7_P	510.3560	689,578 ± 58,089	710,335 ± 73,384	758,848 ± 49,685	733,637 ± 53,343	0.5841	1.3389	0.1217
LysoPC (18:3)	C_26_H_48_NO_7_P	518.3247	620,814 ± 20,714	591,900 ± 19,836	658,460 ± 22,577	675,799 ± 31,645	0.1217	0.3985	1.1643
LysoPC (18:2)	C_26_H_50_NO_7_P	520.3403	6,388,637 ± 262,115	6,025,486 ± 300,959	6,617,717 ± 260,021	6,878,238 ± 274,508	2.2535	4.8170	15.1889
LysoPC (18:1)	C_26_H_52_NO_7_P	522.3560	5,375,808 ± 229,038	5,364,828 ± 250,194	5,593,899 ± 214,117	5,425,134 ± 241,245	4.2976	3.7059	4.2779
LysoPC (18:0)	C_26_H_54_NO_7_P	524.3716	5,661,866 ± 195,118	5,610,496 ± 261,241	5,976,007 ± 255,107	5,830,224 ± 224,489	2.5863	3.3556	5.4463
LysoPC (20:5)	C_28_H_48_NO_7_P	542.3247	771,884 ± 61,530	784,374 ± 53,112	997,008 ± 89,800	876,925 ± 59,723	2.1218	0.4055	1.2595
LysoPC (20:4)	C_28_H_50_NO_7_P	544.3403	2,512,566 ± 118,266	2,294,343 ± 118,562	2,188,892 ± 126,719	1,958,744 ± 83,560	2.9839	3.3959	6.5629
LysoPC (22:6)	C_30_H_50_NO_7_P	568.3403	1,268,789 ± 89,205	1,160,444 ± 72,253	1,322,936 ± 96,792	1,153,481 ± 79,319	1.8183	1.6819	0.2714
SM (d18:0/16:1)	C_39_H_79_N_2_O_6_P	703.5754	918,463 ± 110,095	628,987 ± 81,863	1,046,537 ± 118,533	1,088,608 ± 142,939	0.9346	4.2113	7.9088
Lactosylceramide (d18:1/12:0)	C_42_H_79_NO_13_	806.5630	5,977,825 ± 630,100	8,134,712 ± 703,408	6,832,476 ± 741,718	5,908,934 ± 712,523	4.8718	8.3007	5.9197
^1^PCs			44,523,937 ± 2,514,233	44,154,177 ± 1,968,873	43,454,754 ± 2,366,214	50,137,749 ± 2,118,552			

Mean ± SEM. ^*^ *q* < 0.05, ^**^ *q* < 0.01, ^***^ *q* < 0.001 derived from paired *t*-test. ^*†*^ *q* < 0.05, ^*††*^ *q* < 0.01, ^*†††*^ *q* < 0.001 derived from independent *t*-test in follow-up. ^*‡*^ *q* < 0.05, ^*‡‡*^ *q* < 0.01, ^*‡‡‡*^ *q* < 0.001 derived from changed values between control and whole-grain groups. ^1^PCs were detected by Orbitrap MS; therefore, all detected PC amounts were combined. SM, sphingomyelin

### Correlations among fasting glucose, plasma and PBMC Lp-PLA_2_ activities, biochemical parameters, and major PBMC metabolites

**Fig. 2:**
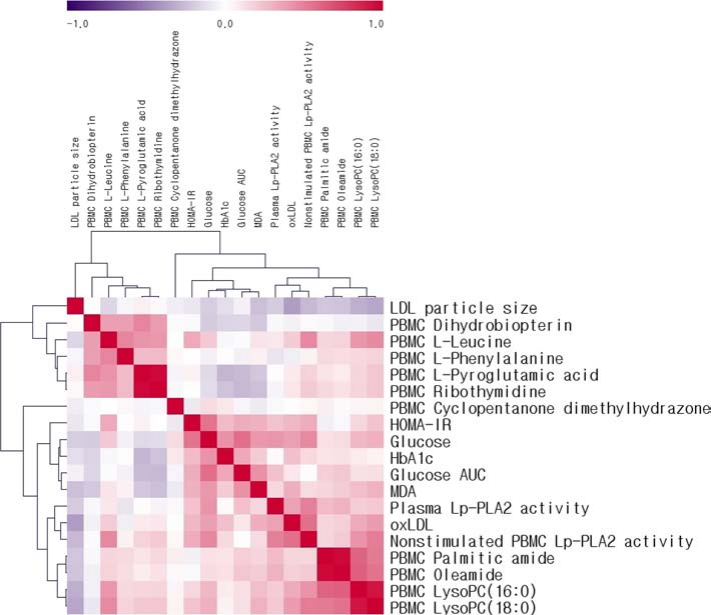
Correlation matrix for changes in biochemical characteristics and PBMC metabolites in total subjects. Supervised hierarchical clustering identifies the most important 10 metabolites and 9 biochemical characteristics. Correlations were obtained by deriving Spearman correlation coefficients. *Red*, positive correlation. *Purple*, negative correlation

The correlation matrix of changes in glucose, Lp-PLA_2_ activities in plasma and PBMC, biochemical parameters, and major PBMC metabolites was computed (Fig. 2). Analysis of metabolic changes including all subjects identified the following correlations: glucose correlated positively with insulin, HOMA-IR, plasma Lp-PLA_2_ activity (*r* = 0.454, *P* < 0.001), MDA, ox-LDL, Lp-PLA_2_ in PBMC (*r* = 0.511, *P* < 0.001), glucose AUC, C-peptide, HbA_1c_, and PBMC lysoPCs after adjusting for age, gender, BMI, smoking, and drinking. After adjusting for confounding variables, plasma Lp-PLA_2_ activity correlated positively with glucose, HOMA-IR, ox-LDL, Lp-PLA_2_ activity in PBMC (*r* = 0.516, *P* < 0.001), glucose AUC, PBMC palmitic amide, and PBMC oleamide. After adjusting for confounding variables, PBMC Lp-PLA_2_ activity correlated positively with glucose, HOMA-IR, plasma Lp-PLA_2_ activity, ox-LDL, PBMC L-leucine, PBMC oleamide, PBMC lysoPCs, and correlated negatively with LDL particle size (Fig. 2).

## Discussion

We identified four PBMC metabolites that had statistically significant differences after dietary intervention, including L-leucine, oleamide, lysoPC (16:0), and lysoPC (18:0); however, there were no significant differences in plasma metabolites after dietary intervention. These aspects of results were also shown in both subjects with prediabetes and T2D, respectively. PBMCs may be a useful tool for nutrigenomics and understanding the pathophysiology of chronic disease due to their active metabolism [14, 15]. These results identify PBMC metabolites as powerful metabolomics tools to detect diet-induced metabolic changes.

Improving glycemic control in the whole-grain group decreases ox-LDL, and reduces PBMC Lp-PLA_2_ activity and PBMC lysoPCs. A strong correlation between PBMC Lp-PLA_2_ activity and ox-LDL, but not LDL-cholesterol, is consistent with a previous report of a direct effect of ox-LDL on Lp-PLA_2_ expression in THP-1 monocytes [16]. Ox-LDL may upregulate PBMC Lp-PLA_2_ expression in smokers [17]. Ox-phospholipids in LDL particles are hydrolyzed by Lp-PLA_2_ at the *sn*-2 position to produce bioactive ox-FFAs and lysoPCs. Only 1 − 5 % of the total non-ox-LDL PC content is lysoPC; however, up to 40 − 50 % of LDL PC is converted to lysoPC during LDL oxidation [18]. This study identified strongly positive correlations among ox-LDL, PBMC Lp-PLA_2_ activity, PBMC lysoPCs, which may indicate that ox-LDL and PBMC Lp-PLA_2_ activity are major determinants of PBMC lysoPC levels.

A negative correlation between PBMC Lp-PLA_2_ activities, PBMC lysoPCs with LDL particle size is consistent with a previous report of Lp-PLA_2_ binding preference for small dense LDL [19]. This study and other work [6] reported positive correlations among glucose, PBMC Lp-PLA_2_ activity, and plasma Lp-PLA_2_ activity. A strongly positive correlation between PBMC and plasma Lp-PLA_2_ activities also is observed in healthy subjects [17, 20]. In a porcine diabetes model, PBMC Lp-PLA_2_ expression is upregulated in the presence of glycation end products [21]. Increases in circulating Lp-PLA_2_ activity and increased ox-LDL levels in hypercholesterolemic pigs are primarily due to plaque macrophages [22]. These results indicate that the primary sources of plasma Lp-PLA_2_ are plaque macrophages [22] and PBMC [17]. This could explain our observations of lower plasma Lp-PLA_2_ activity changes compared with that of PBMC Lp-PLA_2_ activity, and no significant differences in plasma metabolite changes between two groups.

Reduced Lp-PLA_2_ activity in plasma and PBMC in the whole-grain group could be a marker of metabolic changes induced by increased consumption of protein relative to carbohydrate. Diet composition is an important factor in inflammatory processes of blood cells [23]. A study of macronutrient composition determined that increasing dietary protein from 19 % energy intake to 30 % yielded immediate and persistent downregulation of immunological genes in PBMCs [9]. Replacing 5 % of energy from carbohydrates with energy from protein and measured a 2.2 nmol/min/mL reduction in Lp-PLA_2_ activity that was independent of other changes in lipid profiles [5]. Our study replaced 7 % of energy from carbohydrate with approximately 4 % energy from protein and 3 % energy from fat.

Whole grains, legumes, and vegetables contain many antioxidants, vitamins, minerals, and phytochemicals [24, 25]. Antioxidants slow the oxidation rate of reduced substrates [24, 26]. Soybean phytochemicals reduce lipid peroxidation *in vivo* and attenuate LDL oxidation [27]. Our observed changes in glucose and HOMA-IR strongly correlated with changes in MDA and ox-LDL in patients with prediabetes or T2D, consistent with a previous report [28]. We observed positive correlation between changes in HOMA-IR and PBMC L-leucine, but not plasma L-leucine. This may be due to a negligible effect of PBMC L-leucine on plasma L-leucine, or the 12-week dietary intervention may not be long enough to change plasma L-leucine. The whole-grain group also had greater reduction in PBMC oleamide, but not in plasma, compared with control group. We identified PBMC oleamide (VIP = 22.5845) as the most important metabolite for evaluating differences between two groups at the end of the study. Recently, Ha et al. [29] identified plasma oleamide as the most important metabolite for distinguishing nondiabetic from diabetic males. Therefore, positive correlations between changes in PBMC oleamide, Lp-PLA_2_, PBMC palmitic amide, and PBMC lysoPCs observed in our study could be partly due to dietary-induced effects on blood cell inflammatory processes [23].

This study detected many metabolic markers using UPLC-LTQ-Orbitrap MS, but most are currently unidentified. Endogenous biomolecule databases for use with LC-MS − based metabolomics research are still under construction [30]. Despite this limitation, UPLC-LTQ-Orbitrap MS metabolomics and multivariate data analysis identified greater reductions in PBMC L-leucine, PBMC oleamide, PBMC lysoPCs in the whole-grain group than control group; however, there were no significant differences in plasma metabolites between two groups.

## Conclusion

This study demonstrates that replacing refined rice with whole grains and legumes induced greater differences in PBMC Lp-PLA_2_ activity and metabolites than in plasma metabolites in nonobese patients with prediabetes or newly-diagnosed T2D. Therefore, consumption of minimally refined grains, legumes, and vegetables should be recommended to control glucose metabolism and reduce cardiovascular risk factors in patients with IFG, IGT, or newly-diagnosed T2D.

## Abbreviations

Lp-PLA_2_: Lipoprotein-associated phospholipase A_2_
T2D: Type-2 diabetes
PBMC: Peripheral blood mononuclear cell
IFG: Impaired fasting glucose
IGT: Impaired glucose tolerance
BMI: Body mass index
BP: Blood pressure
HDL: High density lipoprotein
LDL: Low density lipoprotein
FFA: Free fatty acid
HbA_1c_: Hemoglobin A_1c_
IR: Insulin resistance
HOMA: Homeostasis-model assessment
FBS: Fetal bovine serum
hs-CRP: High-sensitivity C-reactive protein
MDA: Malondialdehyde
TBARS: Thiobarbituric acid-reactive substances
Ox: Oxidized
MS: Mass spectrometer
PLS-DA: Partial least-squares discriminant analysis
WHR: Waist-hip ratio
AUC: Area under the curve
VIP: Variable important in the projection
